# Vitamin D-binding protein controls T cell responses to vitamin D

**DOI:** 10.1186/s12865-014-0035-2

**Published:** 2014-09-18

**Authors:** Martin Kongsbak, Marina Rode von Essen, Trine Bøegh Levring, Peter Schjerling, Anders Woetmann, Niels Ødum, Charlotte Menné Bonefeld, Carsten Geisler

**Affiliations:** Department of International Health, Immunology and Microbiology, Faculty of Health and Medical Sciences, University of Copenhagen, Copenhagen, Denmark; Department of Orthopedic Surgery M, Institute of Sports Medicine, Bispebjerg Hospital, Copenhagen, Denmark; Center for Healthy Aging, Faculty of Health and Medical Sciences, University of Copenhagen, Copenhagen, Denmark

**Keywords:** T cells, T cell activation, Vitamin D, Vitamin D-binding protein, CYP27B1, Megalin, Cubilin

## Abstract

**Background:**

*In vitro* studies have shown that the active form of vitamin D_3_, 1α,25-dihydroxyvitamin D3 (1,25(OH)_2_D_3_), can regulate differentiation of CD4^+^ T cells by inhibiting Th1 and Th17 cell differentiation and promoting Th2 and Treg cell differentiation. However, the serum concentration of 1,25(OH)_2_D_3_ is far below the effective concentration of 1,25(OH)_2_D_3_ found in *in vitro* studies, and it has been suggested that 1,25(OH)_2_D_3_ must be produced locally from the inactive precursor 25-hydroxyvitamin D3 (25(OH)D_3_) to affect ongoing immune responses *in vivo*. Although it has been reported that activated T cells express the 25(OH)D-1α-hydroxylase CYP27B1 that converts 25(OH)D_3_ to 1,25(OH)_2_D_3_, it is still controversial whether activated T cells have the capacity to produce sufficient amounts of 1,25(OH)_2_D_3_ to affect vitamin D-responsive genes. Furthermore, it is not known how the vitamin D-binding protein (DBP) found in high concentrations in serum affects T cell responses to 25(OH)D_3_.

**Results:**

We found that activated T cells express CYP27B1 and have the capacity to produce sufficient 1,25(OH)_2_D_3_ to affect vitamin D-responsive genes when cultured with physiological concentrations of 25(OH)D_3_ in serum-free medium. However, if the medium was supplemented with serum or purified DBP, DBP strictly inhibited the production of 1,25(OH)_2_D_3_ and 25(OH)D_3_-induced T cell responses. In contrast, DBP did not inhibit the effect of exogenous 1,25(OH)_2_D_3_. Actin, arachidonic acid and albumin did not affect the sequestration of 25(OH)D_3_ by DBP, whereas carbonylation of DBP did.

**Conclusions:**

Activated T cells express CYP27B1 and can convert 25(OH)D_3_ to 1,25(OH)_2_D_3_ in sufficiently high concentrations to affect vitamin D-responsive genes when cultured in serum-free medium. However, DBP sequesters 25(OH)D_3_ and inhibits the production of 1,25(OH)_2_D_3_ in T cells. To fully exploit the immune-regulatory potential of vitamin D, future studies of the mechanisms that enable the immune system to exploit 25(OH)D_3_ and convert it to 1,25(OH)_2_D_3_*in vivo* are required.

**Electronic supplementary material:**

The online version of this article (doi:10.1186/s12865-014-0035-2) contains supplementary material, which is available to authorized users.

## Background

Following antigen recognition CD4^+^ T cells differentiate into one of several types of Th cells including Th1, Th2, Th17 and Treg cells that secrete distinct sets of cytokines [1-3]. Studies have suggested that, in addition to the cytokine milieu, vitamin D is an important determinant in this differentiation of CD4^+^ T cells [4]. Thus, *in vitro* studies have shown that the active form of vitamin D_3_, 1α,25-dihydroxyvitamin D3 (1,25(OH)_2_D_3_), inhibits production of IFN-γ and augment the production of IL-4, thereby limiting Th1 and promoting Th2 cell differentiation [5-9]. Furthermore, 1,25(OH)_2_D_3_ inhibits Th17 cell differentiation and induces differentiation of Treg cells [10-12]. It is therefore generally believed that vitamin D plays an anti-inflammatory role, and accordingly vitamin D deficiency has been associated with increased risk of autoimmune diseases such as type 1 diabetes mellitus [13], lupus erythematosus [14] and multiple sclerosis [15,16].

25-hydroxyvitamin D3 (25(OH)D_3_) is the inactive precursor of 1,25(OH)_2_D_3_ and is considered the best parameter for evaluation of the vitamin D status of a subject. The normal range of serum 25(OH)D_3_ concentrations is 25–170 nM [17]. The serum concentration of the active 1,25(OH)_2_D_3_ is approximately 1000-fold lower (60–110 pM) and far below the effective concentration of 1,25(OH)_2_D_3_ found in *in vitro* studies. Thus, in most *in vitro* studies more than a 100-fold higher concentration of 1,25(OH)_2_D_3_ than found in serum is often required to obtain an effect [7,10-12,18,19]. It has therefore been suggested that the level of circulating 1,25(OH)_2_D_3_ is too low to affect immune responses *in vivo*, and that sufficient levels are obtained by local conversion of 25(OH)D_3_ to 1,25(OH)_2_D_3_ [20]. In accordance, it has been shown that activated antigen presenting cells (APC) express the 25(OH)D-1α-hydroxylase CYP27B1 that converts 25(OH)D_3_ to 1,25(OH)_2_D_3_, and that APC can produce 1,25(OH)_2_D_3_ from 25(OH)D_3_*in vitro* and respond to this through the vitamin D receptor (VDR) in an autocrine fashion [20-23]. Elevated levels of 1,25(OH)_2_D_3_ in association with hypercalcemia have been observed in patients with sarcoidosis, tuberculosis, and other infections and inflammatory diseases in which the pathology is characterized by granuloma formation [24], supporting the hypothesis that activated macrophages can produce significant amounts of 1,25(OH)_2_D_3_*in vivo*.

Like APC, activated T cells express the VDR and CYP27B1 [20,21,25-29]. However, whether T cells can convert 25(OH)D_3_ to 1,25(OH)_2_D_3_ in physiological relevant concentrations and respond to this in an autocrine fashion is a matter of debate. Most studies on the effect of vitamin D on T cells have not addressed this question as they investigated the direct effects of supra-physiological concentrations of 1,25(OH)_2_D_3_ and not how 25(OH)D_3_ affects T cell responses. One study has shown that isolated T cells have the ability to convert 25(OH)D_3_ to 1,25(OH)_2_D_3_ in concentrations that actually affects vitamin D-responsive genes in an autocrine fashion [21]. In agreement, we recently found that purified CD4^+^ T cells have the ability to produce substantial amounts of 1,25(OH)_2_D_3_ when activated in the presence of 25(OH)D_3_ [27]. In contrast, another recent study found that although activated T cells do express CYP27B1, the expression level is not sufficiently high to allow production of 1,25(OH)_2_D_3_ in concentrations that affect vitamin D-responsive genes [20]. The authors found that 25(OH)D_3_ only affected T cell responses when APC were present, and suggested that APC locally secrete sufficient amounts of 1,25(OH)_2_D_3_ to directly influence the surrounding T cells in a paracrine fashion.

Other important players influencing the bioavailable levels of vitamin D are the vitamin D-binding protein (DBP) and albumin. 25(OH)D_3_ and 1,25(OH)_2_D_3_ circulate bound to DBP (85–90%) and albumin (10–15%) with less than 1% in their free form [30,31]. Studies of DBP knock-out mice have shown that DBP acts as a vitamin D reservoir by protecting 25(OH)D_3_ and 1,25(OH)_2_D_3_ from degradation and renal secretion [32]. However, DBP also sequesters 25(OH)D_3_ and 1,25(OH)_2_D_3_ and inhibits their action on monocytes, DC and keratinocytes *in vitro* [20,33,34]. How DBP affects T cell responses to 25(OH)D_3_ still needs to be determined.

The objectives of this study were to further elucidate whether T cells have the ability to convert 25(OH)D_3_ to 1,25(OH)_2_D_3_ in proportions that affect a panel of vitamin D-responsive genes in an autocrine fashion and to investigate how DBP regulates T cell responses to 25(OH)D_3_.

## Results

### Activated T cells express CYP27B1 and have the capacity to convert 25(OH)D_3_ to 1,25(OH)_2_D_3_

**Figure 1:**
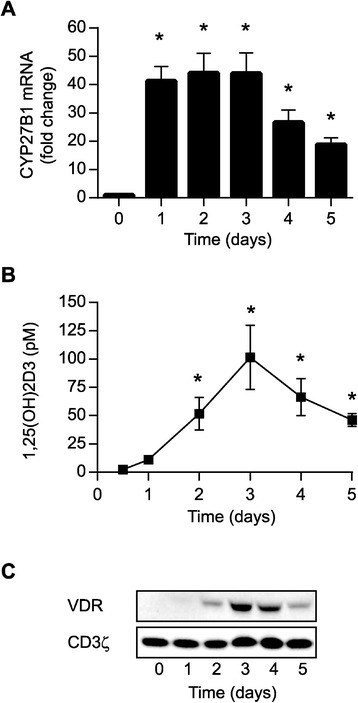
**Activated T cells express CYP27B1 and have the capacity to convert 25(OH)D**_**3**_ **to 1,25(OH)**_**2**_ **D**_**3**_ **. (A)** Relative CYP27B1 mRNA expression in T cells activated for 0–5 days normalized to CYP27B1 expression in naïve T cells. Values are given as mean + SEM from 3 independent experiments, *p < 0.05. **(B)** 1,25(OH)_2_D_3_ in the medium of T cells activated for 0–5 days in the presence of 100 nM 25(OH)D_3_. Data are given as mean ± SEM from 3 independent experiments, *p < 0.05. **(C)** Representative Western blot of VDR and CD3ζ (loading control) expression in T cells activated for 0–5 days.

In order to convert 25(OH)D_3_ to 1,25(OH)_2_D_3_ cells must express the 25(OH)D-1α-hydroxylase CYP27B1. To determine whether naïve CD4^+^ T cells express CYP27B1, we purified CD45RA^+^CD4^+^ cells from the blood of healthy donors. The resulting cell population contained 95–98% CD4^+^ T cells of which more than 96% were CD45RA^+^ (Additional file 1: Figure S1). The purified cells were stimulated with CD3/CD28 beads for 0–5 days in serum-free medium and their expression of CYP27B1 mRNA was subsequently measured. We found that naïve CD4^+^ T cells express no or very low levels of CYP27B1. However, the cells started to express CYP27B1 mRNA shortly after stimulation, and the expression peaked after 2–3 days of stimulation (Figure 1A). These results suggested that activated human CD4^+^ T cells have the capacity to convert 25(OH)D_3_ to 1,25(OH)_2_D_3_. To validate this, we stimulated purified CD4^+^ T cells in the presence of 100 nM 25(OH)D_3_ corresponding to physiological concentrations of 25(OH)D_3_ in serum and then measured the production of 1,25(OH)_2_D_3_. We found that activated CD4^+^ T cells produced 1,25(OH)_2_D_3_ with a kinetic similar to the kinetics of CYP27B1 expression in the cells, and that the production peaked after 3 days of stimulation (Figure 1B). Finally, to determine whether the cells expressed the receptor for 1,25(OH)_2_D_3_, we determined the expression of the VDR in CD4^+^ T cells stimulated for 0–5 days. We found that VDR expression peaked simultaneously with the peak production of 1,25(OH)_2_D_3_ at day 3 (Figure 1C). Taken together, these experiments demonstrated that activated human CD4^+^ T cells express CYP27B1, that they have the capacity to convert 25(OH)D_3_ at physiological concentrations to the active 1,25(OH)_2_D_3_, and that they express the receptor for 1,25(OH)_2_D_3_.

### Activated T cells have the ability to produce 1,25(OH)_2_D_3_ in sufficiently high concentrations to affect vitamin D-responsive genes

**Figure 2:**
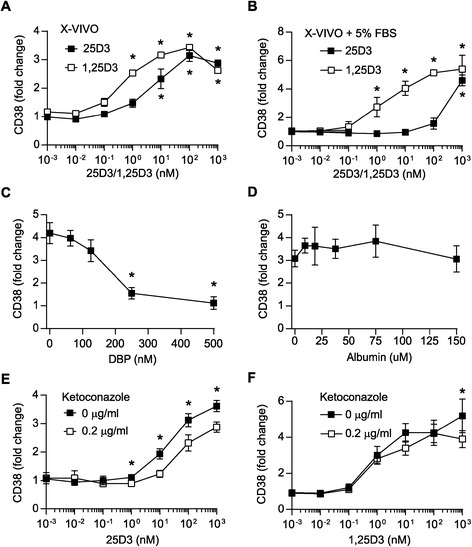
**In the absence of serum activated T cells can produce 1,25(OH)**_**2**_ **D**_**3**_ **in concentrations that affect CD38 expression.** Relative CD38 cell surface expression on T cells activated for 3 days in X-VIVO 15 **(A)** or X-VIVO 15 supplemented with 5% FBS **(B)** in the presence of the indicated concentrations of 25(OH)D_3_ and 1,25(OH)_2_D_3_ normalized to CD38 cell surface expression on T cells activated in the absence of 25(OH)D_3_ and 1,25(OH)_2_D_3_. Relative CD38 cell surface expression on T cells activated for 3 days in X-VIVO 15 medium supplemented with increasing concentrations of DBP **(C)** or albumin **(D)** in the presence of 100 nM 25(OH)D_3_ normalized to CD38 cell surface expression on T cells activated in the absence of 25(OH)D_3_. Relative CD38 cell surface expression on T cells activated for 3 days in the absence or presence of ketoconazole and the indicated concentrations of 25(OH)D_3_ **(E)** or 1,25(OH)_2_D_3_ **(F)** normalized to CD38 cell surface expression on T cells activated in the absence of 25(OH)D_3_ or 1,25(OH)_2_D_3_. **(A-F)** Mean values ± SEM from 3 independent experiments are plotted, *p < 0.05.

Having demonstrated that activated CD4^+^ T cells have the capacity to produce the active form of vitamin D and that they simultaneously express the VDR, we next asked whether the production of 1,25(OH)_2_D_3_ was sufficiently high to induce autocrine activation of vitamin D-responsive genes. To study this, we took advantage of the finding that the cell surface expression levels of CD38 on activated T cells are dependent on the concentration of 1,25(OH)_2_D_3_ present during T cell activation [12,35]. Consequently, we activated CD4^+^ T cells in serum-free medium in the presence of increasing concentrations of 25(OH)D_3_ or 1,25(OH)_2_D_3_ and then measured CD38 expression at day 3. In accordance with previous studies, we found that 1,25(OH)_2_D_3_ significantly up-regulated CD38 expression in activated T cells in a dose-dependent manner (Figure 2A) [12,35]. In addition, we found that 25(OH)D_3_ also induced CD38 up-regulation, suggesting that activated T cells can produce sufficiently high concentrations of 1,25(OH)_2_D_3_ to affect vitamin D-responsive genes (Figure 2A). We noted that whereas 25(OH)D_3_ at a physiological concentration of 100 nM induced maximum CD38 up-regulation, 1,25(OH)_2_D_3_ was required at a 100-fold higher concentration (10 nM) than physiological levels (60–110 pM) to induce the same effect. That a supra-physiological concentration of 1,25(OH)_2_D_3_ was required to obtain an effect in our study is in good line with previous studies [7,10-12,18,19]. However, in contrast to our observations that 25(OH)D_3_ at 100 nM had a clear effect on T cells, most previous studies on 25(OH)D_3_ required at least a 100-fold higher concentration of 25(OH)D_3_ than found in serum to induce an effect [21]. One explanation for this discrepancy could be that whereas our experiments were performed in serum-free medium most previous studies have been performed in medium supplemented with serum. To study whether the presence of serum could explain the discrepancy between the concentrations of 25(OH)D_3_ required to obtain an effect on T cells, we activated CD4^+^ T cells in the presence of increasing concentrations of 25(OH)D_3_ and 1,25(OH)_2_D_3_ in medium supplemented with 5% fetal bovine serum (FBS) and measured CD38 expression at day 3. We found that the presence of serum significantly shifted the concentration of 25(OH)D_3_ required to induce CD38 expression (Figure 2B). Thus, compared to serum-free medium approximately 100-fold higher concentrations of 25(OH)D_3_ were required to induce a similar effect on CD38 expression in T cells cultured in medium supplemented with serum. In contrast, the presence of serum did not significantly shift the concentration of 1,25(OH)_2_D_3_ required to induce CD38 expression. To separately analyse the role of DBP and albumin in this serum-mediated inhibition of the effect of 25(OH)D_3_ on T cells, we activated CD4^+^ T cells in the presence of 100 nM 25(OH)D_3_ in serum-free medium supplemented with increasing concentrations of either DBP or albumin and measured CD38 expression at day 3. We found that DBP at concentrations above 250–500 nM completely abolished the effect of 25(OH)D_3_ on CD38 expression, whereas albumin did not affect the effect of 25(OH)D_3_ (Figure 2C and D).

Although T cells converted 25(OH)D_3_ to 1,25(OH)_2_D_3_ when activated in serum-free medium, we could not exclude the possibility that the observed 25(OH)D_3_-induced up-regulation of CD38 was caused directly by 25(OH)D_3_ and not by 1,25(OH)_2_D_3_. To exclude this possibility, we determined the effect of the CYP27B1 inhibitor ketoconazole on CD38 up-regulation. We activated T cells in the absence or presence of ketoconazole and increasing concentrations of 25(OH)D_3_. We found that ketoconazole efficiently inhibited 25(OH)D_3_-induced CD38 up-regulation (Figure 2E). These observations indicated that conversion of 25(OH)D_3_ to 1,25(OH)_2_D_3_ is required for 25(OH)D_3_-induced CD38 up-regulation. If this is the case and ketoconazole does not in itself influence CD38 expression then ketoconazole should not affect 1,25(OH)_2_D_3_-induced CD38 up-regulation. Consequently, we activated T cells in the absence or presence of ketoconazole and increasing concentrations of 1,25(OH)_2_D_3_. We found that ketoconazole did not affect 1,25(OH)_2_D_3_-induced CD38 up-regulation (Figure 2F). These experiments indicated that it is not 25(OH)D_3_ itself that induces CD38 up-regulation, but that the conversion of 25(OH)D_3_ to 1,25(OH)_2_D_3_ is required for 25(OH)D_3_-induced CD38 up-regulation.

Thus, we could conclude that activated T cells have the capacity to produce sufficient 1,25(OH)_2_D_3_ to affect vitamin D-responsive genes as measured by CD38 expression when cultured with physiological concentrations of 25(OH)D_3_ in serum-free medium. However, addition of DBP to the medium inhibited the effect of 25(OH)D_3_. In contrast, DBP did not seem to affect the concentration of 1,25(OH)_2_D_3_ required to regulate vitamin D-responsive genes.

### DBP inhibits 25(OH)D_3_-induced T cell responses

**Figure 3:**
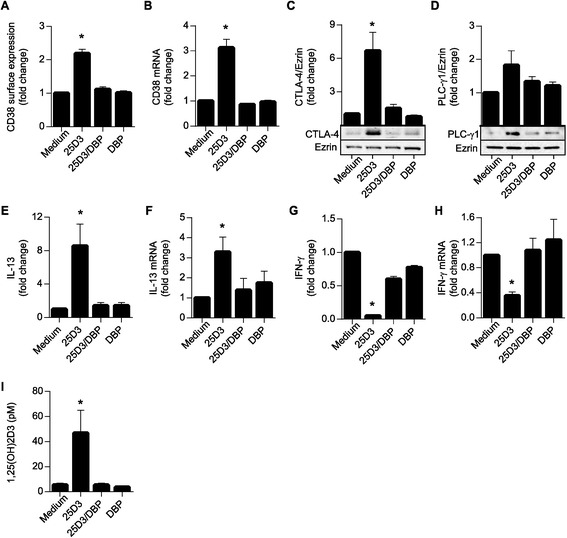
**DBP limits the bioavailability of 25(OH)D**_**3**_ **and inhibits vitamin D-induced gene expression in T cells.** Relative CD38 cell surface expression (n = 6) **(A)** and mRNA expression (n = 4) **(B)**, CTLA-4 expression (n = 4) **(C)**, PLC-γ1 expression (n = 4) **(D)**, IL-13 production (n = 3) **(E)** and mRNA expression (n = 4) **(F)**, IFN-γ production (n = 4) **(G)** and mRNA expression (n = 4) **(H)**, and 1,25(OH)_2_D_3_ in the medium (n = 6) **(I)** of T cells activated for 3 days in X-VIVO 15 (medium), or X-VIVO 15 supplemented with either 100 nM 25(OH)D_3_ (25D3), 1 μM DBP (DBP) or the combination of 25(OH)D_3_ and DBP (25D3/DBP). The mean concentration of IL-13 and IFN-γ in the supernatants of cells activated in X-VIVO 15 (medium) was 246 pg/ml and 26 ng/ml, respectively. **(A-I)** Mean + SEM from the numbers of independent experiments indicated by n in the legend above.

To determine whether the inhibitory effect of DBP on 25(OH)D_3_ was limited to CD38 gene expression or was a general phenomenon for vitamin D-responsive genes in T cells, we studied the influence of 25(OH)D_3_ and DBP alone or in combination on the expression of molecules known to be encoded by vitamin D-responsive genes such as CTLA-4 [7,20], PLC-γ1 [25,36], IL-13 [37] and IFN-γ [7] in addition to CD38 [35]. We stimulated CD4^+^ T cells for 3 days in serum-free medium or serum-free medium supplemented with either 25(OH)D_3_ or DBP alone or 25(OH)D_3_ plus DBP. We confirmed that 25(OH)D_3_ up-regulates CD38 mRNA and protein in activated T cells, and that this up-regulation is completely inhibited by DBP (Figure 3A and B). Likewise, we found that the 25(OH)D_3_-induced up-regulation of CTLA-4, PLC-γ1 and IL-13 and down-regulation of IFN-γ was abolished by DBP (Figure 3C-H). Thus, DBP generally inhibits the effect of 25(OH)D_3_ on vitamin D-responsive genes in T cells. To further elucidate the underlying mechanism behind the inhibition of 25(OH)D_3_ in T cells mediated by DBP, we measured the production of active 1,25(OH)_2_D_3_ in the medium from the cultures described above. We found that in the presence of 25(OH)D_3_ activated T cells produced significant amounts of 1,25(OH)_2_D_3_; however, addition of DBP completely abolished the production of 1,25(OH)_2_D_3_ (Figure 3I). Thus, we could conclude that DBP strongly constrains the effect of 25(OH)D_3_ on vitamin D-responsive genes in T cells by inhibiting the conversion of 25(OH)D_3_ to 1,25(OH)_2_D_3_.

### T cells take up DBP from the environment by macropinocytosis

**Figure 4:**
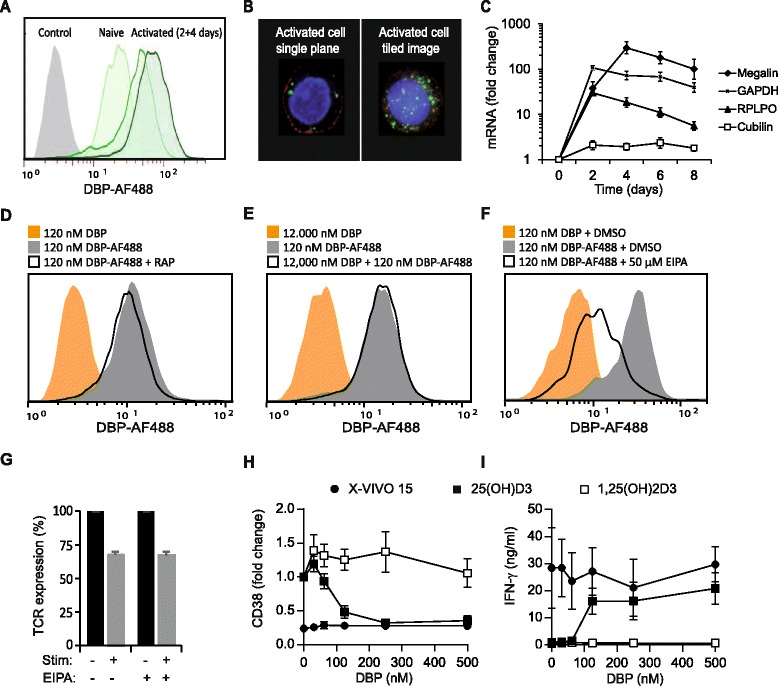
**T cells take up DBP from the environment by macropinocytosis. (A)** Representative flow cytometry histograms showing DBP-AF488 fluorescence of naïve T cells and T cells activated for 2 and 4 days and subsequently incubated with 120 nM DBP-AF488 for 12 h at 37°C. **(B)** Representative confocal microscopy images illustrating the uptake of DBP-AF488 in activated T cells. DBP-AF488 are shown in green; cell membrane (TCR) in red; nucleus in blue (DAPI stain). **(C)** Relative megalin, cubilin, GAPDH and RPLP0 mRNA expression in T cells activated from 0–8 days normalized to the respective mRNA expression in naïve T cells. (D-F) Flow cytometry histograms illustrating DBP-AF488 fluorescence of activated T cells incubated with 120 nM DBP-AF488 in the absence or presence of **(D)** RAP (1 μM), **(E)** unlabelled DBP (12,000 nM), or **(F)** EIPA (50 μM). **(G)** TCR internalization in activated T cells re-stimulated with 30 nM PDBu for 60 min in the absence or presence of EIPA (50 μM). TCR surface expression of non-stimulated cells was set to 100%. **(H-I)** CD38 expression **(H)** and IFN-γ production **(I)** of T cells activated for 3 days with the indicated concentrations of DBP in X-VIVO 15 and X-VIVO 15 supplemented with either 25(OH)D_3_ (100 nM) or 1,25(OH)_2_D_3_ (10 nM). **(C, G-I)** Data are shown as mean ± SEM from 3 independent experiments.

DBP is internalized by megalin-mediated endocytosis in kidney and mammary cells that express megalin and cubilin [38-40]. Megalin-mediated endocytosis of DBP facilitates uptake and conversion of 25(OH)D_3_ to 1,25(OH)_2_D_3_ in these types of cells [39,40]. To study whether T cells can take up DBP from the environment, we incubated naïve and activated T cells with DBP conjugated to Alexa Fluor 488 (DBP-AF488). Flow cytometry revealed increased fluorescence of activated T cells compared to naïve T cells, suggesting that activated T cells take up DBP-AF488 (Figure 4A). To exclude that the increased fluorescence simply was caused by DBP-AF488 adhering to the cell surface of the activated T cells as suggested in a previous study [41], we analysed the cells by confocal microscopy. We found that DBP-AF488 resided in small vesicles in the cytosol and could thus conclude that activated T cells actually take up DBP from the medium (Figure 4B). To determine whether this uptake might be mediated by megalin, we measured megalin and cubilin mRNA expression in naïve and activated T cells. Activated T cells up-regulated megalin mRNA to a level more than 100-fold higher than in naive T cells, whereas the cubilin mRNA levels were low in both naïve and activated T cells (Figure 4C). In kidney and mammary cells megalin-mediated endocytosis of DBP is, in addition to megalin, dependent on the presence of cubilin at the cells surface [38,40]. To determine whether DBP was taken up by megalin-mediated endocytosis in activated T cells despite their low expression of cubilin, we incubated cells with DBP-AF488 in the absence or presence of the megalin antagonist receptor-associated protein (RAP) as previously described [33,38,39]. We found that RAP did not influence DBP-AF488 uptake in the T cells (Figure 4D). Furthermore, blocking antibodies against megalin and calcium deprivation, known to inhibit megalin-mediated endocytosis [39], did not affect DBP uptake (Additional file 2: Figure S2). In line with this, high concentrations of DBP did not affect the uptake of DBP-AF488 (Figure 4E). Taken together these experiments indicated that DBP is not taken up by megalin-mediated endocytosis in T cells.

In addition to receptor-mediated endocytosis, cells can take up extracellular molecules by macropinocytosis. Macropinocytosis is characterized by its augmentation by cell activation and that it can be inhibited by amiloride [42,43]. We found that treatment of the cells with the amiloride analogue 5-(N-Ethyl-N-isopropyl)-amiloride (EIPA) significantly inhibited uptake of DBP-AF488 (Figure 4F). To ensure that EIPA only blocked macropinocytosis and not receptor-mediated endocytosis, we measured the effect of EIPA on T cell receptor (TCR) internalization in parallel. TCR internalization is dependent on binding of the CD3γ di-leucine based internalization motif to adaptor molecules of clathrin-coated pits [44,45] and hence should not be influenced by EIPA. We found that EIPA did not affect TCR internalization (Figure 4G). Thus, we could conclude that DBP uptake by CD4^+^ T cells is not mediated by megalin-mediated endocytosis but most likely by macropinocytosis.

Whereas megalin-mediated endocytosis of DBP facilitates uptake and conversion of 25(OH)D_3_ to 1,25(OH)_2_D_3_ in kidney and mammary cells [39,40], macropinocytosis of DBP-25(OH)D_3_ complexes did certainly not facilitate conversion of 25(OH)D_3_ to 1,25(OH)_2_D_3_ in T cells (Figure 3). To study whether DBP inhibited the effects of 1,25(OH)_2_D_3_ in the same way as it inhibited the effects of 25(OH)D_3_ on T cells, we stimulated CD4^+^ T cells for 3 days in the presence of either 25(OH)D_3_ or 1,25(OH)_2_D_3_ and increasing concentrations of DBP. After 3 days we measured CD38 expression and IFN-γ production. We found that in the absence of DBP both 25(OH)D_3_ and 1,25(OH)_2_D_3_ up-regulated CD38 expression and down-regulated IFN-γ production. However, whereas addition of DBP clearly inhibited the effect of 25(OH)D_3_ it did not influence the effect of 1,25(OH)_2_D_3_ on T cell responses (Figure 4H and I).

### Neither actin, arachidonic acid nor albumin affect the DBP-mediated inhibition of 25(OH)D_3_

**Figure 5:**
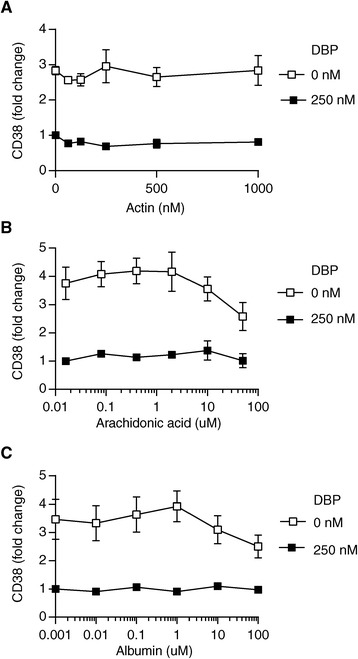
**Neither actin, arachidonic acid or albumin increases the bioavailability of 25(OH)D**_**3**_ **for T cells.** Relative CD38 cell surface expression on T cells activated for 3 days in the presence of 25(OH)D_3_ (100 nM) and in the absence or presence of DBP (250 nM) and the indicated concentrations of **(A)** actin, **(B)** arachidonic acid or **(C)** albumin. Mean values ± SEM from 3 independent experiments are plotted.

In addition to 25(OH)D_3_ and 1,25(OH)_2_D_3_, DBP can bind actin [46,47] and fatty acids [48,49]. It has been suggested that binding of fatty acids to DBP decreases the affinity of DBP for 25(OH)D_3_ [48,49], and in theory this could increase the bioavailability of 25(OH)D_3_ for immune cells. To study whether binding of actin or arachidonic acid to DBP affected DBP-mediated inhibition of 25(OH)D_3_-induced T cell responses, we stimulated CD4^+^ T cells in the absence or presence of DBP plus 25(OH)D_3_ and increasing concentrations of actin or arachidonic acid. We found that neither actin nor arachidonic acid affected 25(OH)D_3_-induced T cell responses as measured by CD38 expression (Figure 5A and B). Since albumin binds 25(OH)D_3_ with lower affinity than DBP, the ratio between DBP and albumin could in theory also affect the bioavailability of 25(OH)D_3_; however, as for actin and arachidonic acid we found that albumin did not affect the DBP-mediated inhibition of 25(OH)D_3_-induced T cell responses (Figure 5C).

### DBP carbonylation impedes DBP-mediated inhibition of 25(OH)D_3_-induced T cell responses

**Figure 6:**
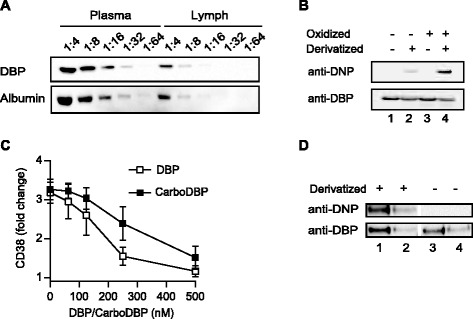
**DBP carbonylation impedes DBP-mediated inhibition of 25(OH)D3-induced CD38 expression. (A)** Western blot of DBP and albumin in serial dilutions of plasma and central lymph. **(B)** Western blot of non-oxidized (lanes 1 and 2) and oxidized (lanes 3 and 4) DBP that subsequently had been derivatized with 2,4-dinitrophenyl hydrazine to detect carbonyl groups (lanes 2 and 4) or left untreated (lanes 1 and 3). The membranes were first analysed with anti-DNP antibodies to detect carbonylated DBP. Subsequently, the membranes were stripped and re-blotted with anti-DBP antibodies to detect total DBP. **(C)** Relative CD38 cell surface expression on T cells activated in the presence of 25(OH)D_3_ (100 nM) and the indicated concentrations of DBP or carbonylated DBP (CarboDBP). Mean values ± SEM from 3 independent experiments are plotted. **(D)** Representative Western blot analysis of carbonylated DBP and total DBP (loading control) in human serum. Precipitated DBP was either derivatized with 2,4-dinitrophenyl hydrazine to detect carbonyl groups (lanes 1 and 2) or left untreated (lanes 3 and 4). Lanes 1 and 3 present precipitated DBP diluted 1:4, and lanes 2 and 4 present precipitated DBP diluted 1:8.

The experiments above demonstrated that activated T cells have the capacity to convert 25(OH)D_3_ to 1,25(OH)_2_D_3_ given that 25(OH)D_3_ is available in a sufficiently high free concentration but that DBP normally binds and sequesters 25(OH)D_3_. The concentration of DBP relative to 25(OH)D_3_ determines the ratio of free to sequestered 25(OH)D_3_ [50]. The concentration of DBP in serum is approximately 5 μM, i.e. 50–100 fold higher than the concentration of 25(OH)D_3_, and the free to sequestered ratio of 25(OH)D_3_ is very low [17,50,51]. However, primary immune responses are most often initiated in secondary lymphoid organs like lymph nodes where the concentration of DBP is unknown. It has been reported that the protein concentration in extracellular fluid/peripheral lymph might be as low as 5–10% of that found in serum [52,53], suggesting that the concentration of DBP in secondary lymphoid organs might be significantly lower than the concentration in serum. In agreement, by semi-quantitative analyses we found that the concentration of DBP in central lymph from mini-pigs was approximately 25% of the concentration of DBP in serum (Figure 6A).

Another mechanism that might decrease the concentration of functional DBP and thereby increase the availability of 25(OH)D_3_ is carbonylation of DBP. Carbonylation is a protein modification induced by oxidative stress [54], and increased carbonylation of serum proteins including DBP is seen during inflammatory responses [55-57]. Carbonylation of DBP could lead to a higher concentration of free 25(OH)D_3_ due to a reduced affinity of carbonylated DBP for 25(OH)D3 and/or increased degradation of DBP. To investigate this, we activated CD4^+^ T cells in the presence of 25(OH)D_3_ and increasing concentrations of either unmodified or carbonylated DBP (Figure 6B). We found that carbonylated DBP did not inhibit the effect of 25(OH)D_3_ to the same extent as unmodified DBP as measured by CD38 expression (Figure 6C). Thus, inflammation-induced carbonylation of DBP might contribute to an increased availability of 25(OH)D_3_ during an immune reaction.

Interestingly, we observed that a fraction of the non-oxidized purified human DBP was actually carbonylated (Figure 6B, lane 2). This suggested that carbonylated DBP is found in human serum as recently described for rat serum [55]. To investigate whether carbonylated DBP is found in human serum we immunoprecipitated DBP from freshly drawn human blood and either derivatized it with 2,4-dinitrophenyl hydrazine to detect carbonylated DBP or left it untreated before Western blot analysis with anti-DNP antibodies. We found that carbonylated DBP is found in human serum (Figure 6D).

## Discussion

This study shows that activated human CD4^+^ T cells express CYP27B1 and produce sufficient amounts of 1,25(OH)_2_D_3_ to affect vitamin D-responsive genes when cultured in the presence of physiological concentrations of 25(OH)D_3_ in DBP/serum-free medium.

We found that CYP27B1 expression becomes strongly up-regulated in activated CD4^+^ T cells (Figure 1A), and our results thereby confirm and extend previous reports on CYP27B1 expression in T cells [20,21,28,29]. However, although activated T cells express CYP27B1, is has been discussed whether they actually have the ability to convert 25(OH)D_3_ to 1,25(OH)_2_D_3_. Thus, some studies have found that activated T cells can convert 25(OH)D_3_ to 1,25(OH)_2_D_3_ [21,27,28], whereas a recent study found that T cells do not have this ability [20]. By measuring 1,25(OH)_2_D_3_ in the medium of T cells activated in the presence of 25(OH)D_3_ we found that CYP27B1 expressed by the T cells is functional, and that T cells have the ability to produce significant amounts of 1,25(OH)_2_D_3_ (Figure 1B). When determining the capacity of T cells to convert 25(OH)D_3_ to 1,25(OH)_2_D_3_ the kinetics of CYP27B1 expression is important to take into account. We and others [28] found that 1,25(OH)_2_D_3_ production is very low 24 hours after T cell activation but that it strongly increases 48 hours after activation. We find it plausible that the missing detection of 1,25(OH)_2_D_3_ produced by activated T cells in the study by Jeffery et al. [20] was due to the fact that the authors measured 1,25(OH)_2_D_3_ production after only 24 hours of activation. Thus, our study clarifies that activated human CD4^+^ T cells have the capacity to convert 25(OH)D_3_ to 1,25(OH)_2_D_3_. Furthermore, we demonstrated that activated T cells have the capacity to produce significantly high amounts of 1,25(OH)_2_D_3_ to affect vitamin D-responsive genes such as CD38 [35], CTLA-4 [7,20], PLC-γ1 [25,36], IL-13 [37] and IFN-γ [7] (Figures 2 and 3).

Despite the ability of activated T cells to convert 25(OH)D_3_ to 1,25(OH)_2_D_3_, addition of DBP to the medium inhibited the effect of 25(OH)D_3_ on vitamin D-responsive genes in a dose-dependent manner (Figures 2, 3, 4). Interestingly, DBP did not seem to significantly inhibit 1,25(OH)_2_D_3_-induced T cell responses (Figure 4). The affinity of DBP for 25(OH)D_3_ is significantly higher than for 1,25(OH)_2_D_3_ with a K_d_ of 1.4 nM and 25 nM, respectively [30,31], and this could be one of the reasons that DBP sequestered 25(OH)D_3_ more efficiently than 1,25(OH)_2_D_3_. Megalin-mediated endocytosis of DBP facilitates uptake and conversion of 25(OH)D_3_ to 1,25(OH)_2_D_3_ in some types of cells such as renal proximal tubule cells and mammary epithelial cells [39,40]. We found that activated T cells express megalin and take up DBP. However, they do not take up DBP by megalin-mediated endocytosis as demonstrated by the lack of effect of RAP, blocking anti-megalin antibodies and competition experiments (Figure 4 and Additional file 2: Figure S2). In line with this, previous studies have demonstrated that megalin-mediated endocytosis of DBP is dependent of the co-expression of cubilin [38], and we found that cubilin expression was very low in naïve T cells and that it was not up-regulated following T cell activation. Interestingly, we found that EIPA, which inhibits macropinocytosis, reduced the DBP up-take. Thus, activated T cells take up DBP, but this up-take is not mediated by megalin-mediated endocytosis but most likely by macropinocytosis. In contrast to megalin-mediated endocytosis that promotes the conversion of 25(OH)D_3_ to 1,25(OH)_2_D_3_ in kidney and mammary cells [39,40], macropinocytosis of 25(OH)D_3_-DBP did not deliver 25(OH)D_3_ for subsequent conversion to 1,25(OH)_2_D_3_ in T cells. Similar results have been found for monocytes that also take up DBP by a megalin-independent mechanism and where DBP inhibits the conversion of 25(OH)D_3_ to 1,25(OH)_2_D_3_ [33].

By titrations of 25(OH)D_3_ and 1,25(OH)_2_D_3_ in serum-free medium, we found that maximal effect on vitamin D-regulated genes was obtained at 100 and 10 nM, respectively, as assessed by CD38 expression (Figure 2A). Addition of serum or purified DBP considerably shifted the concentration of 25(OH)D_3_ but not of 1,25(OH)_2_D_3_ required to affect vitamin D-responsive genes (Figure 2B). These results support that the physiological concentration of 1,25(OH)_2_D_3_ (60–110 pM) is not sufficiently high to affect T cell responses, and that a significant local production of 1,25(OH)_2_D_3_ is essential to reach concentration (>1000 pM) required to affect T cells as previously suggested [20]. Furthermore, these results indicate that mechanisms must exist whereby 25(OH)D_3_ is released from DBP and becomes available for the conversion to 1,25(OH)_2_D_3_, given that 25(OH)D_3_ affects T cell responses *in vivo*. In a search for such mechanisms, we investigated whether actin, arachidonic acid or albumin affected the sequestration of 25(OH)D_3_ by DBP, as DBP can bind actin [46,47] and fatty acids [48,49], and such binding might affect the affinity of DBP for 25(OH)D_3_ [48,49]. However, neither actin, arachidonic acid nor albumin affected the DBP-mediated inhibition of 25(OH)D_3_-induced T cell responses (Figure 5). Local concentrations and/or modifications of DBP might also affect the availability of 25(OH)D_3_ to T cells. Inflammation-induced oxidative stress can result in oxidative modifications of proteins leading to protein carbonylation [54-56]. Protein carbonylation is irreversible and leads to disturbances in protein conformation and function [54]. Interestingly, we found evidence that carbonylation of DBP impedes DBP-mediated inhibition of 25(OH)D_3_-induced T cell responses (Figure 6). Thus, inflammation-induced oxidative stress could locally lead to DBP carbonylation and thereby to a higher concentration of free 25(OH)D_3_. Finally, the DBP gene is polymorphic, and the three most common DBP isotypes termed GC1S, GC1F and GC2 have varying affinities for 25(OH)D_3_, which might also influence the availability and conversion of 25(OH)D_3_ to 1,25(OH)_2_D_3_ and thereby the efficiency of 25(OH)D_3_-induced T cell responses [20,58].

Experiments by nature indicate that significant amounts of 1,25(OH)_2_D_3_ actually can be produced locally by the involved immune cells during inflammation/infection *in vivo*. Thus, elevated systemic levels of 1,25(OH)_2_D_3_ can be observed in patients with granulomatous diseases such as sarcoidosis and tuberculosis [24]. The granulomas are characterized by a central area of activated macrophages surrounded by activated CD4^+^ T cells. This suggests that interactions between activated T cells and macrophages might induce mechanisms that allow efficient conversion of 25(OH)D_3_ to 1,25(OH)_2_D_3_*in vivo* despite the presence of DBP. This is in good accordance with previous studies which found that treatment of macrophages with IFN-γ or soluble CD40L increases their expression of CYP27B1 and their capacity to convert 25(OH)D_3_ to 1,25(OH)_2_D_3_ [59-61]. Thus, whether vitamin D actually affects a given T cell response *in vivo* probably relies on a mixture of factors in addition to the concentration of 25(OH)D_3_ such as the isotype, local concentration and degradation rate of DBP and the expression levels of CYP27B1, the VDR and the 1,25(OH)_2_D_3_-24-hydroxylase CYP24A1 of the cells locally involved in the immune response.

## Conclusions

In summary, activated T cells express CYP27B1 and can convert 25(OH)D_3_ to 1,25(OH)_2_D_3_ in sufficiently high concentrations to affect vitamin D-responsive genes when cultured in serum-free medium. However, DBP sequesters 25(OH)D_3_ and inhibits the production of 1,25(OH)_2_D_3_ in T cells. To fully exploit the immune-regulatory potential of vitamin D, further studies of the mechanisms that enable the immune system to exploit 25(OH)D_3_ and convert it to 1,25(OH)_2_D_3_ are required.

## Methods

### Chemicals

25(OH)D_3_ (BML-DM100-0001) and 1,25(OH)_2_D_3_ (BML-DM200-0050) were from Enzo Life Sciences, Inc., Ann Arbor, MI. Stock solutions of 2.5 mM 25(OH)D_3_ and 2.4 mM 1,25(OH)_2_D_3_ were prepared in anhydrous (≥99.5%) ethanol and stored at −80°C. To determine 1,25(OH)_2_D_3_ in the medium we used the 1,25-Dihydroxy Vitamin D EIA kit (AC-62F1) from IDS, Tyne and Wear, UK according to the manufacturer’s instructions. DBP (A50674H) and albumin (A8763) purified from human serum were from Meridian Life Sciences and Sigma-Aldrich, respectively. Actin (A2522), arachidonic acid (A9673) and ketoconazole (K1003) were from Sigma-Aldrich. Serum and central lymph from mini-pigs were provided by the Department for Experimental Medicine, University of Copenhagen, Denmark.

### Cell culture

Mononuclear cells from blood were isolated by Lymphoprep (Axis-Shield, Oslo, Norway) density gradient centrifugation from healthy donors after obtaining informed, written consent in accordance with the Declarations of Helsinki principles for research involving human objects. The study was approved by the local Ethics Committee (H-3-2009-132, The Committees of Biomedical Research Ethics for the Capital Region in Denmark). Naïve CD4^+^ T cells were isolated, cultured and activated as previously described [27]. The cells were activated for 3 days in serum-free X-VIVO 15 medium (1041, Lonza, Verviers, Belgium) if not otherwise stated.

### Flow cytometry

For flow cytometry analyses of CD38 and TCR expression the cells were stained with anti-CD38-APC (HIT2) or anti-CD3ε-PE (UCHT1) both from BD Biosciences and analyzed on a FACS Calibur. Fold change in CD38 surface expression was calculated as mean CD38 fluorescence intensity of cells stimulated in the presence of 25(OH)D_3_/1,25(OH)_2_D_3_ divided with mean CD38 fluorescence intensity of cells stimulated in the absence of 25(OH)D_3_/1,25(OH)_2_D_3_. Percent TCR surface expression was calculated as (mean fluorescence intensity of stimulated cells divided with mean fluorescence intensity of untreated cells) × 100%.

### Western blot and ELISA

Western blot analyses were performed as previously described [27,62]. Following incubation with primary antibody, the membranes were washed, and the proteins visualized following 60 min incubation at room temperature with HRP-conjugated rabbit anti-mouse Ig, swine anti-rabbit Ig or rabbit anti-goat Ig (P0260, P0399 and P0449 DAKO, Glostrup, Denmark) using ECL (Amersham Biosciences) technology. The primary antibodies used were anti-VDR, anti-CD3ζ, anti-CTLA-4 and anti-albumin (D-6, 6B10.2, C-19 and F-8, Santa Cruz Biotecnology), anti-PLC-γ1 (05–163, Upstate Biotechnology), anti-ezrin (3145, Cell Signaling Technology) and anti-DBP (SAB2501100, Sigma Aldrich). For band density quantification ECL exposed sheets were analysed in a ChemiDoc MP Imaging System from Bio-Rad. Measurement of the cytokines IL-13 and IFN-γ were determined by ELISA according to the manufacturer’s protocol (Ready-Set-Go; eBioscience).

### Real-time RT-PCR

mRNA for CYP27B1, CD38, IL-13, IFN-γ, megalin, cubilin, GAPDH and RPLP0 were measured by real-time RT-PCR as previously described [27]. Primers used (sense/antisense primer) were:

CYP27B1: (AAGCGCAGCTGTATGGGGAGAC/GCTCAGGCTGCACCTCAAAATG),

CD38: (CTGGAGAAAGGACTGCAGCAACAA/GCATCACATGGACCACATCACA),

IL-13: (GATTCTGCCCGCACAAGGTCTC/GTAAGAGCAGGTCCTTTACAAACTGGG),

IFN-γ: (CAGCTCTGCATCGTTTTGGGTTC/CCATTATCCGCTACATCTGAATGACCT).

Megalin: (CATGAGGTGTGCAATGGTGTGG/TCTGTACAAGGTTTAGGGGTCGGTT).

Cubilin: (GGTCCTCTTGACTTTTGTGTCCTTCC/CATCGTTGACACAGCTTCCCGT).

GAPDH: (CCTCCTGCACCACCAACTGCTT/GAGGGGCCATCCACAGTCTTCT).

RPLP0; (GGAAACTCTGCATTCTCGCTTCCT/CCAGGACTCGTTTGTACCCGTTG).

The data were normalized to number of cells by calculation from the total RNA yield per cell in each sample (the raw data represents number of target cDNA molecules measured per 12.5 ng total RNA).

### DBP uptake and TCR internalization

For studies of cellular uptake of DBP purified DBP was conjugated with Alexa Fluor 488 (AF488) using a commercial kit (A10235, Molecular Probes). 120 nM DBP-AF488 was added to cell cultures of 1 × 10^6^ cells/ml X-VIVO 15 for 12 h at 37°C. The cells were subsequently washed and analyzed by flow cytometry. Samples incubated with 120 nM non-conjugated DBP were included as controls. In some studies the cells were activated for 3 days, washed and resuspended in X-VIVO 15 including 120 nM DBP-AF488 and either 1 μM RAP (Merch Millipore), 20 μg/ml anti-megalin blocking Ab (anti-Megalin Ab, C-19 and H-245 from Santa Cruz Biotechnology), 4 μM EGTA for calcium deprivation, 12,000 nM non-conjugated DBP to outcompete possible specific uptake of DBP-AF488 by receptor-mediated endocytosis or 50 μM 5-(N-ethyl-N-isopropyl)-amiloride (EIPA) (Sigma Aldrich) an inhibitor of macropinocytosis [43]. For experiments including EIPA, DMSO was added to all samples. The cells were subsequently analysed by flow cytometry. For microscopy, cells were incubated with DBP-AF488 for 12 h at 37°C and then stained with anti-CD3 (UCHT1, BD) followed by an AlexaFluor568 coupled anti-mouse Ig and DAPI (nuclear staining). The cells were fixed in 1% paraformaldehyde and analysed by confocal microscopy (Nikon TE 2000-E). For TCR down-regulation experiments the activated cells were rested for 24 h after removal of the CD3/CD28 beads. Hereafter, the cells were adjusted to 1 × 10^6^ cells/ml, pre-treated with either DMSO or 50 μM EIPA dissolved in DMSO for 30 min and then treated with 30 nM phorbol 12,13-dibutyrate (PDBu) (Sigma-Aldrich) for 60 min as previously described [63,64]. The TCR surface expression levels were subsequently determined by flow cytometry.

### DBP carbonylation and immunoprecipitation

For carbonylation of DBP, 1 mg purified DBP was oxidized in 100 μl oxidation buffer (50 mM Hepes, 100 mM KCl, 10 mM MgCl_2_, pH 7.4). An additional 100 μl oxidation buffer including 50 mM ascorbic acid and 200 μM FeCl_3_ was added and the tube incubated for 15 h at 37°C with shaking. 1 mM EDTA in oxidation buffer was added to stop the reaction. The solution was transferred to a VivaSpin500 column (VS0122, Sautorius Stedim Biotech) and the buffer changed to PBS (column was spun down once with PBS/1 μM EDTA and twice with PBS). To test the efficiency of the carbonylation reaction, Western blot analyses were performed comparing non-oxidized and oxidized DBP (CarboDBP) after derivatization with 2,4-dinitrophenyl hydrazine using the commercial Oxyblot Protein Oxidation Detection kit (S7150, Millipore) according to the manufacturer’s instruction. To determine whether carbonylated DBP is found in human serum, we isolated DBP from freshly isolated human serum by classical immunoprecipitation [65,66] using anti-DBP antibodies and protein A coated beads. The precipitated DBP was either derivatized with 2,4-dinitrophenyl hydrazine or left untreated before Western blot analyses with anti-DNP antibodies to detect carbonylated DBP. The membranes were subsequently stripped and re-blotted with anti-DBP antibodies to detect total DBP.

### Statistical analysis

Statistical analyses were performed using Student’s *t* test with a 5% significance level, unpaired and paired observations and equal variance.

## Additional files

Additional file 1: Figure S1. Flow cytometric analysis of (A) PBMC and (B) purified naive CD4+ T cells. Additional file 2: Figure S2. T cells do not take up DBP by megalin-mediated endocytosis. Flow cytometry histograms illustrating DBP-AF488 fluorescence of activated T cells incubated with 120 nM DBP or 120 nM DBF-AF488 in the absence or presence of (A) EGTA (4 μM), (B) anti-megalin anti-body (20 μg/ml) or (C) EGTA (4 μM) plus anti-megalin antibody (20 μg/ml).

## Abbreviations

1,25(OH)_2_D_3_: 1α,25-dihydroxyvitamin D3
25(OH)D_3_: 25-hydroxyvitamin D3
VDR: Vitamin D receptor
DBP: Vitamin D-binding protein
APC: Antigen presenting cell
TCR: T cell receptor
FBS: Fetal bovine serum
RAP: Receptor-associated protein
EIPA: 5-(N-Ethyl-N-isopropyl)-amiloride
PDBu: Phorbol 12,13-dibutyrate
