# Supplementary material for: Vitamin D-binding protein controls T cell responses to vitamin D
Source: BMC Immunol. 2014 Sep 18;15:35. doi: 10.1186/s12865-014-0035-2 (PMC4177161; doi:10.1186/s12865-014-0035-2)
Supplement: Additional file 1: Figure S1. — Flow cytometric analysis of (A) PBMC and (B) purified naive CD4+ T cells. [file 12865_2014_35_MOESM1_ESM.pdf]

Additional file 1: Figure S1

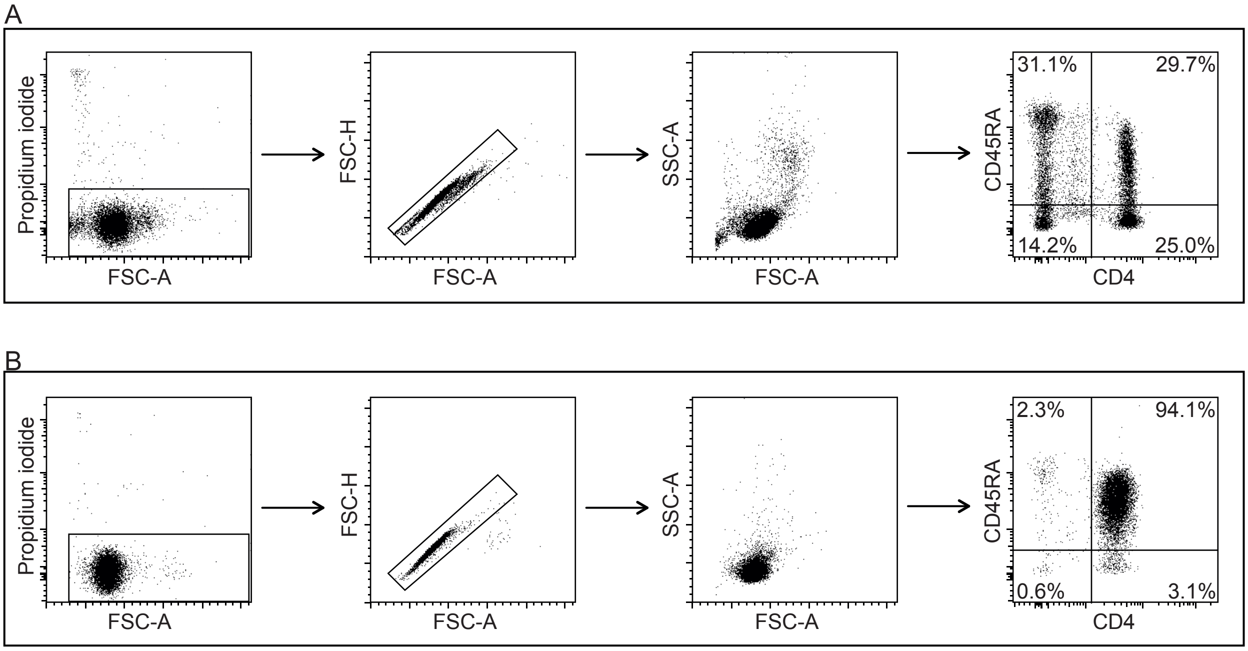

Additional file 1: Figure S1

Flow cytometric analysis of (A) PBMC and (B) purified naive CD4+ T cells.
