# Supplementary material for: Vitamin D-binding protein controls T cell responses to vitamin D
Source: BMC Immunol. 2014 Sep 18;15:35. doi: 10.1186/s12865-014-0035-2 (PMC4177161; doi:10.1186/s12865-014-0035-2)
Supplement: Additional file 2: Figure S2. — T cells do not take up DBP by megalin-mediated endocytosis. Flow cytometry histograms illustrating DBP-AF488 fluorescence of activated T cells incubated with 120 nM DBP or 120 nM DBF-AF488 in the absence or presence of (A) EGTA (4 μM), (B) anti-megalin anti-body (20 μg/ml) or (C) EGTA (4 μM) plus anti-megalin antibody (20 μg/ml). [file 12865_2014_35_MOESM2_ESM.pdf]

Additional file 2: Figure S2

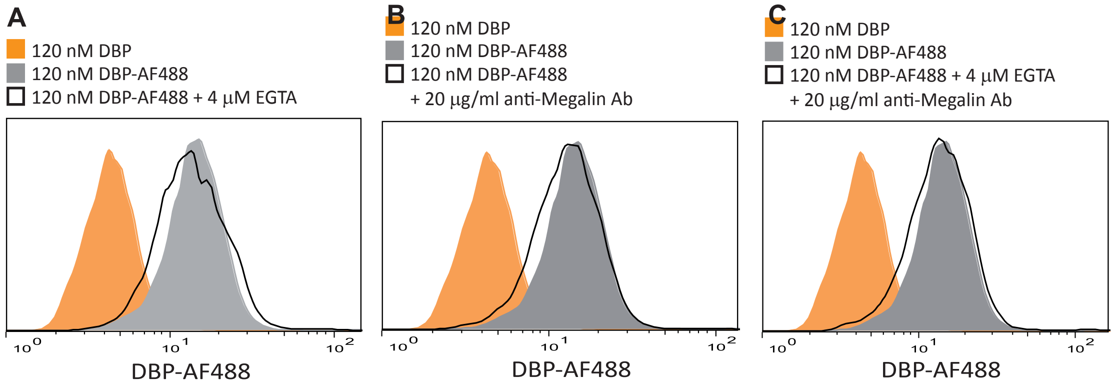

Additional file 2: Figure S2 T cells do not take up DBP by megalin-mediated endocytosis
Flow cytometry histograms illustrating DBP-AF488 fluorescence of activated T cells incubated with 120 nM DBP or 120 nM DBF-AF488 in the absence or presence of (A) EGTA (4 µM), (B) anti-megalin anti-body (20 µg/ml) or (C) EGTA (4 µM) plus anti-megalin antibody (20 µg/ml).
